# Digital health and Clinical Patient Management System (CPMS) platform utility for data sharing of neuromuscular patients: the Italian EURO-NMD experience

**DOI:** 10.1186/s13023-023-02776-5

**Published:** 2023-07-21

**Authors:** Fernanda Fortunato, Francesca Bianchi, Giulia Ricci, Francesca Torri, Francesca Gualandi, Marcella Neri, Marianna Farnè, Fabio Giannini, Alessandro Malandrini, Nila Volpi, Diego Lopergolo, Vincenzo Silani, Nicola Ticozzi, Federico Verde, Davide Pareyson, Silvia Fenu, Silvia Bonanno, Vincenzo Nigro, Cristina Peduto, Paola D’Ambrosio, Roberta Zeuli, Mariateresa Zanobio, Esther Picillo, Serenella Servidei, Guido Primiano, Cristina Sancricca, Monica Sciacco, Roberta Brusa, Massimiliano Filosto, Stefano Cotti Piccinelli, Elena Pegoraro, Tiziana Mongini, Luca Solero, Giulio Gadaleta, Chiara Brusa, Carlo Minetti, Claudio Bruno, Chiara Panicucci, Valeria A. Sansone, Christian Lunetta, Alice Zanolini, Antonio Toscano, Alessia Pugliese, Giulia Nicocia, Enrico Bertini, Michela Catteruccia, Daria Diodato, Antonio Atalaia, Teresinha Evangelista, Gabriele Siciliano, Alessandra Ferlini

**Affiliations:** 1 Medical Genetics Unit, Department of Medical Sciences, University of Ferrara, Ferrara, Italy; 2 Medical Genetics Unit, Department of Mother and Child, Sant’Anna University Hospital of Ferrara, Ferrara, Italy; 3 Department of Clinical and Experimental Medicine, Neurological Clinic, University of Pisa, Pisa, Italy; 4 Department of Medical, Surgical and Neurological Sciences, University of Siena, Siena, Italy; 5 Department of Neurology and Laboratory of Neuroscience, IRCCS Istituto Auxologico Italiano, Milan, Italy; 6 Department of Pathophysiology and Transplantation, “Dino Ferrari” Center, Università Degli Studi Di Milano, Milan, Italy; 7 Unit of Rare Neurodegenerative and Neurometabolic Diseases, Fondazione IRCCS Istituto Neurologico Carlo Besta, Milan, Italy; 8 Neuroimmunology and Neuromuscular Diseases Unit, Fondazione IRCCS Istituto Neurologico Carlo Besta, Milan, Italy; 9 Department of Precision Medicine, University of Campania “L. Vanvitelli”, Naples, Italy; 10 Telethon Institute of Genetics and Medicine (TIGEM), Pozzuoli, Naples, Italy; 11 Fondazione Policlinico Universitario Agostino Gemelli IRCCS, Rome, Italy; 12 Neuromuscular and Rare Disease Unit, Department of Neuroscience, Foundation IRCCS Ca’ Granda Ospedale Maggiore Policlinico, Milan, Italy; 13 Department of Clinical and Experimental Sciences, University of Brescia, Brescia, Italy; 14 ASST Spedali Civili Di Brescia, Brescia, Italy; 15 NeMO-Brescia Clinical Center for Neuromuscular Diseases, Brescia, Italy; 16 Department of Neuroscience, University of Padova, Padua, Italy; 17 Department of Neurosciences “Rita Levi Montalcini”, University of Turin, Turin, Italy; 18 Pediatric Neurology Unit and Muscle Unit, IRCCS Istituto Giannina Gaslini, Department of Neurosciences, Rehabilitation, Ophthalmology, Genetics, Maternal and Child Health, University of Genoa, Genoa, Italy; 19 Center of Translational and Experimental Myology, IRCCS Istituto Giannina Gaslini, Department of Neurosciences, Rehabilitation, Ophthalmology, Genetics, Maternal and Child Health, University of Genoa, Genoa, Italy; 20 The NEMO (NEuroMuscular Omniservice) Clinical Center, Milan, Italy; 21 Neurorehabilitation Unit, University of Milan, Milan, Italy; 22 Neurology and Neuromuscular Diseases Unit, Department of Clinical and Experimental Medicine, University of Messina, Messina, Italy; 23 ERN-NMD Center of Messina, Department of Clinical and Experimental Medicine, University of Messina, Messina, Italy; 24 Unit of Neuromuscular and Neurodegenerative Disorders, Department of Neurosciences, Bambino Gesù Children’s Hospital IRCCS, Rome, Italy; 25 Service of Neuromyology, APHP-GH Pitié-Salpêtrière, Sorbonne Université, Paris, France; 26 Neuromuscular Morphology Unit, Institute of Myology, GHU Pitié-Salpêtrière, Sorbonne Université, Paris, France

**Keywords:** Telemedicine, CPMS, ERN, Rare diseases, Digital health

## Abstract

**Background:**

The development of e-health technologies for teleconsultation and exchange of knowledge is one of the core purposes of European Reference Networks (ERNs), including the ERN EURO-NMD for rare neuromuscular diseases. Within ERNs, the Clinical Patient Management System (CPMS) is a web-based platform that seeks to boost active collaboration within and across the network, implementing data sharing. Through CPMS, it is possible to both discuss patient cases and to make patients’ data available for registries and databases in a secure way. In this view, CPMS may be considered a sort of a temporary storage for patients’ data and an effective tool for data sharing; it facilitates specialists’ consultation since rare diseases (RDs) require multidisciplinary skills, specific, and outstanding clinical experience.

Following European Union (EU) recommendation, and to promote the use of CPMS platform among EURO-NMD members, a twelve-month pilot project was set up to train the 15 Italian Health Care Providers (HCPs). In this paper, we report the structure, methods, and results of the teaching course, showing that tailored, ERN-oriented, training can significantly enhance the profitable use of the CPMS.

**Results:**

Throughout the training course, 45 professionals learned how to use the many features of the CPMS, eventually opening 98 panels of discussion—amounting to 82% of the total panels included in the EURO-NMD. Since clinical, genetic, diagnostic, and therapeutic data of patients can be securely stored within the platform, we also highlight the importance of this platform as an effective tool to discuss and share clinical cases, in order to ease both case solving and data storing.

**Conclusions:**

In this paper, we discuss how similar course could help implementing the use of the platform, highlighting strengths and weaknesses of e-health for ERNs. The expected result is the creation of a “map” of neuromuscular patients across Europe that might be improved by a wider use of CPMS.

**Supplementary Information:**

The online version contains supplementary material available at 10.1186/s13023-023-02776-5.

## Background

Between 5000 and 8000 rare diseases (RDs) afflict the daily life of more than 30,000,000 people in Europe [1]. They represent a challenge both for patients and for their doctors, who have to tackle highly disabling and poorly known diseases.

In an effort for strengthening the fight against complex diseases by easing diagnostic processes and equalizing therapeutic approaches, in 2017 the European Commission created 24 European References Networks (ERNs), each focusing on a specific group of rare or low-prevalence diseases. ERNs are networks involving national Health Care Providers (HCPs) across Europe [2], as part of a broader strategy to make the national and European health systems more efficient, accessible and resilient. There are 24 ERNs involving 25 European countries, more than 300 hospitals with over 900 HCP units covering all major disease groups so far [3].

Amongst these 24 ERNs, EURO-NMD, European Reference Network for the thematic grouping of rare neuromuscular diseases (NMDs), unites 84 of Europe’s leading NMD clinical and research centres in 25 Member States and includes highly active patients’ organizations [4].

NMDs represent a heterogenous group of more than 400 diseases with a very broad phenotypic spectrum, collectively affecting an estimation of 500,000 EU citizens overall. Although each neuromuscular disease is relatively uncommon (most NMDs show prevalence rates that range between 1 and 10 per 100,000 population) collectively, cause a significant socioeconomic burden of disease [5].Though each ERN focuses on a specific set of diseases, HCPs belonging to different ERNs can easily interface with each other. In these networks, medical specialists across different disciplines are connected in a sort of “virtual” advisory board through a dedicated IT platform and telemedicine tools.

Telemedicine is increasingly used as an everyday approach to facilitate diagnosis and treatment of an heterogeneous set of clinical conditions, from mild to critical [6].

As shown by the COVID-19 pandemics, telemedicine is critical to follow up and keep patients’ assistance over extended periods. The improvement of telemedicine has already changed medical assistance, mainly for those patients with poor mobility due to high disabling diseases [7].

Still, the expertise for diagnosis and management available at specific HCP sites may not be sufficient to achieve the full cohort of care and therapies for all patients, resulting in an increasing request for cross-border e-health technologies providing means for teleconsulting and exchanging knowledge across medical experts [8]. According to the ERNs mission, RD-expertise can be offered by some HCPs, especially for ultra-rare conditions [9]. Periodic meetings and boards are instrumental for networks maintenance, but one of the most outstanding ERN telemedicine tools is the Clinical Patient Management System (CPMS). The CPMS is a digital platform where clinicians belonging to different HCPs can discuss about clinical cases in a secure way and across all ERNs [10]. Using the CPMS, it is possible for health professionals to discuss about patients by sharing images, clinical reports and organizing video meeting where clinicians can discuss *vis à vis*. The CPMS is one of the most fundamental ERN core tasks: reducing health care inequalities within the European Union (EU) by providing access to expert specialized care to all patients with rare and complex diseases. The access to the CPMS is restricted to ERN members and enforces multiple steps of authentication, ensuring that case discussions are fully secure and respectful of patients’ privacy [11].

In short, case discussions happen through the opening of so-called “panels”, where the clinician requiring a medical consultation provides a detailed description of clinical case. Data sharing (*e.g.* radiological images, exams reports, laboratory findings) via panels is possible, so that advice on virtual consultation can be easily provided by the specialists invited both using the chat tool and via video meetings. An outcome that summarizes all steps of case discussion and all conclusive remarks is produced, and patients’ data is available to be used in databases and registries, should the patient agree in giving consent.

In this view, CPMS is more than just a platform for case solving: thanks to its ability to store a high amount of data, it is a real digital database, effective for research and data analysis. It may be considered as an electronic health record/database that keeps all patients data and increases the evidence-based knowledge of rare diseases. It achieves ultra-rare disease sharing and accurate diagnostic as well, and facilitates access to genetic testing and other biochemical testing. Since any functional (clinical, phenotypic, imaging, biochemical, genetic, etc.) study carried out can be reported in the platform, it may serve as a source of functional data providing unprecedented scientific insights as well. Therefore, this growing database will potentially be adopted to support data interpretation as those related to Variant of Uncertain Significance (VUS) [12].

The outstanding added value of CPMS is the capability to let HCPs from all ERNs crosstalk and share cases, therefore establishing a multidisciplinary clinical and diagnostic context, where achieving the correct diagnosis is maximized.

Notwithstanding its potential in improving the quality of health care provision, the CPMS is seldom used to its full potential. As is the case with other telemedicine tools, the relatively steep learning curve, and the inherent complexity of the CPMS system may hamper its use as an everyday tool. These challenges are ever more prominent in the COVID-19 era, when telemedicine became a staple of medical practices [13]. A less than optimal use of the CPMS might be contributed by the lack of a sufficiently detailed knowledge on its correct use and even by the language barrier represented by the digital system that needs to be in English so to allow crosstalk across Europe.

This paper focuses on the CPMS use within the neuromuscular diseases ERN (EURO-NMD), reporting structure, methods and outcome of a twelve-month CPMS hands-on training tailored to healthcare professionals in 15 Italian HCPs part of the EURO-NMD.

Aims of this paper are several: (i) to show that tailored, ERN-oriented, hands-on trainings can significantly enhance the profitable use of the CPMS; (ii) to demonstrate that the use of CPMS platform for ERN EURO-NMD network in Italy is feasible and with potential to be implemented in the work routine; (iii) to elucidate that the wider use of CPMS among Italian HCPs appears as a resource to create evidence and reach oversight on progress made in Europe.

Based on a multi-HCPs virtual teaching course, we also underline added value, positive aspects, and possible implementations which may facilitate wide spreading of CPMS use across ERNs.

## Methods

The consent process for patients was established by DG SANTE in collaboration with the legal and ethical working group of the ERNs. A common information and consent form, complying with General Data Protection Regulation (GDPR), was translated in 24 EU languages and is available to be used across all ERNs.

The local ethical committee approval for each participating centre is not mandatory since the CPMS has obtained a European centralised ethical approval, and therefore EU countries participating to CPMS automatically accepted this approval.

The informed consent process for patients was led by healthcare professionals (geneticists, neurologists, pediatric neuropsychiatrists) who are responsible for enrolling patients in CPMS. The healthcare professional explained CPMS contents, uses, and ethical issues during routine consultation and illustrated the consent form which is composed of three “sessions”: (i) consent to share de-identified data in ERN(s) for care to discuss the case in CPMS (obligatory); (ii) consent to include de-identified data in one or more ERN databases or registries (optional); (iii) consent to be contacted about research in the future (optional).

The English version of the consent form is included in supplementary data (Additional file 1).

### Training setup

The CPMS training course was led by two Italian national medical doctors (MDs), a geneticist and a neurologist. Originally meant to take place in person, the course was then converted in an online training due to the COVID-19 pandemics that disrupted national and international travels for the most part of 2020 and 2021. The Emilia-Romagna Region Lepida platform was used for the entire training [14]. All participants could follow the entire training thanks to screen sharing from either the trainers or the other participants. Prior the course onset, the two MD trainers underwent a teaching course remotely with a CPMS expert from EURO-NMD, who fully disclosed the platform’s functionalities and followed the trainers step-by-step in gaining full control of the CPMS platform. After the teaching course ended, the training course started. Details related to organization and structure of the training course are included in supplementary data (Additional file 2).

### Data analysis and impact assessment

At the end of the training, indicator data on the EURO-NMD platform was collected and compared with the situation prior the course. Indicators chosen include: (i) number of panels, (ii) status of the opened panels.

## Results

The training involved 15 HCPs in Italy (13 in the first part, 15 in the second part), the whole of the national HCPs involved in the EURO-NMD. Forty-five professionals joined the training, of which 27 women and 18 men. Sixteen were Professors, six were residents, three were biologists and 20 were medical doctors. The most represented residency was neurology, but genetics and pediatric neuropsychiatry were also represented. Only four of the participants of the course had CPMS login credentials at the beginning of the course.

**Fig. 1:**
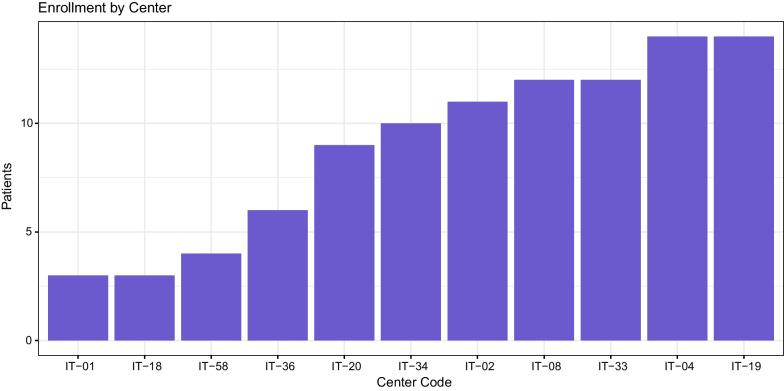
Number of opened panels for each HCP at end of the course (June 2021). HCPs are reported by codes on the x-axis, and number of panels (and therefore number of patients uploaded) is reported on the y-axis. IT-19 University Hospital St. Anna, Ferrara; IT-04 Center for Neuromuscular Diseases, Unit of Neurology, ASST “Spedali Civili”, Brescia; IT-33 Fondazione Policlinico Universitario A. Gemelli, Rome; IT-08 Azienda Ospedaliero-Universitaria Pisana, U.O.C. Neurologia, Pisa; IT-02 University of Campania “Luigi Vanvitelli”—University Hospital “Luigi Vanvitelli”, Naples; IT-34 IRCCS Ca’ Granda Ospedale Maggiore Policlinico, Milan; IT-20 Azienda Ospedaliera Universitaria Città della Salute e della Scienza di Torino, Turin; IT-36 Fondazione IRCCS, Istituto Neurologico C. Besta, Milan; IT-58 Bambino Gesù Children’s Research Hospital IRCCS, Rome; IT-18 Azienda Ospedaliera Universitaria Senese, Siena; IT-01 University of Messina, Messina

At the training onset (September 2020), the total number of panels opened in the EURO-NMD were 23, of which seven from Italian HCPs. During the training, the number of panels increased substantially so that at the end of the course (June 2021) was of 120, of which 98 opened by the Italian branch of EURO-NMD as a direct consequence of the course (81.7% of total panels of EURO-NMD). Throughout the whole training course, panels were opened by 11 of the 15 HCPs that have participated to the course, with different proficiency across different HCPs. On average, each Italian HCP involved in the course opened 8.9 panels (SD = 4.2) (Fig. 1).

**Fig. 2:**
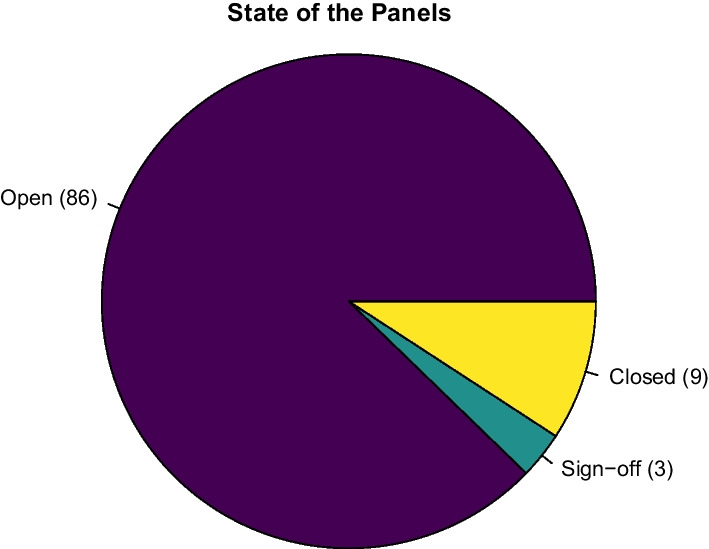
State of panels’ progression after the end of the course (June 2021). State of panels’ after the end of the course is represented in the pie chart showing that 12 panels had completed the CPMS progression timeline

Of the 98 panels newly opened during the training, most of the generated panels were at the “open” step that is the first of the pipeline. Three panels were at the “sign off” step, while nine were “closed”, meaning that 12 panels had completed the CPMS progression timeline (Fig. 2).

**Fig. 3:**
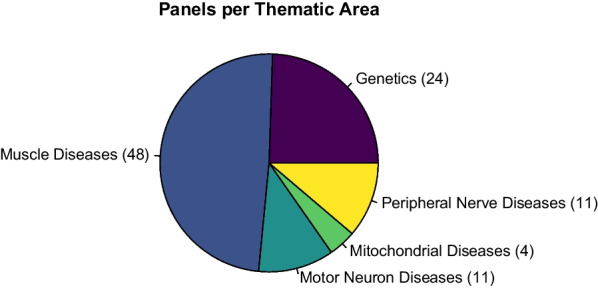
Number of panels for thematic area after the end of the course (June 2021). A considerable difference in numbers of created panels for thematic area is showed in the pie chart

At the end of the course, there was a considerable difference in numbers of created panels for each thematic area. Most of the panels (48) referred to muscle diseases, followed by genetics (24), motor neuron disease (11), peripheral nerve disease (11), and mitochondrial disease (4) (Fig. 3).

Panels were opened regarding diagnosis of patients with two main themes: (i) patients without a known diagnosis or (ii) patients with or without a diagnosis, but with a genetic Variant of Uncertain Significance. Panels were also opened to discuss treatments of patients.

Time spent on each panel was extremely variable depending on: (i) the complexity of each clinical case; (ii) the possible upload of imaging or phenotype-related documentation; (iii) the possible organization of video meetings; (iv) a possible delay between questions and answers from panels’ members.

An average time of approximately 30 min to complete the consultation form, of 30–60 min to carry out video meetings, and of 30 min to upload imaging or phenotype-related documentation was reported by participants.

## Discussion

Among the several tools used by ERNs for networks maintenance, the Clinical Patient Management System (CPMS) allows virtual clinical consultations for complex cases among international groups of top-level experts addressing to one of the most important missions of ERNs: cross-border transfer of knowledge and expertise without physical movement of a patient.

Although the patients may greatly benefit from a thorough implementation of the CPMS, previous reports indicate a less-than-optimal usage of the platform in several ERNs [8, 15]. This was not different in the EURO-NMD, that prior the training object of this report had 23 panels uploaded on the system.

The hands-on training that we had designed started from the most basic functions of the CPMS (including registration and login) and followed through until the most advanced functions of panel discussion. This strategy was successful in increasing the number of registered users on the platform among the participants to the course from four to 32 and in multiplying by a factor five the number of panels eventually opened (Fig. 1). Differences in HCP proficiency in the use of the system and differences in number of patients available are reflected by the number of panels that had been opened across different centers, going from three to 14 (Fig. 1).

We cannot provide numeric data about those patients’ who have been already discussed and diagnoses/treatments already achieved. Indeed, since only 12 panels were closed/signed-off, being this a minority of all discussed cases (98), it is not possible to drive general numerical or data quality conclusions about the outcomes of our CPMS panels.

As far as we know, this is the first hands-on training designed to promote the wider use of the CPMS, therefore our results cannot be compared to already existing literature data [8, 15].

Throughout the course, all HCPs managed to open their own panels and exercised in enrolling patients and scheduling video meetings about complex clinical cases.

Informal feedback collected at the end of the training showed that participants improved their CPMS knowledge. 100% of participants (n = 45) agreed in considering it a powerful diagnostic tool with potential to be implemented in their work routine. The training was intended to be performed in person, yet the COVID-19 pandemics forced us to work remotely. This limitation, however, was convenient in further disclosing the CPMS potential. Video meetings allowed for quick interactions between clinicians also without face-to-face meetings. The strengthening of the use of the CPMS resulted in a strengthening of the Italian HCPs network, especially for the youngest members who had the possibility to discuss a clinical case in a secure online environment with a multidisciplinary team of experts. During the course, attendees directly experienced the easiness of data sharing through the platform and might hand-touched the possibility of securely record patients’ data and cases’ discussions, the latter also instrumental for a teaching purpose.

The training course was also an opportunity to identify criticalities for the CPMS usage as expressed by involved HCPs. These criticalities, seemingly trivial, may have a strong attrition effect on the widespread use of the platform. One of the prominent challenges emerged during the training was the somewhat complex operation of the many functions offered by the platform, a condition common to other ERNs [8, 15]*.*

However, in the initial approach to the platform, technical difficulties experienced by Italian HCPs were quite varied and included: (i) lack of clarity of the several steps needed to access the platform; (ii) complexity of the sections of the consultation form of the CPMS; (iii) inconsistent performance of the email notification system, that in some cases did not reach the intended recipients; (iv) video meeting tool not accessible from some of the HCPs. Notably, some of the issues with the CPMS were traced to limitations imposed on the local networks of the HCPs and required interaction with local ICT teams to be solved. Notably, during the training course, the CPMS platform underwent a major update with changes resulting in a simplification of some sections, allowing a more intuitive utilization of the platform.

It is worth mentioning that some ERN-provided services, CPMS included, are not covered by any financial instruments. Therefore, the CPMS becomes essentially a voluntary and “out of working hours” activity, requiring extra time allocated to its continuous use; this can easily translate in the under-usage of the CPMS platform, substantially hampering the long-term sustainability of the tool.

Besides these aforementioned issues, the wider use of CPMS among Italian HCPs appears an opportunity with high potential but also as a resource to create evidence and reach oversight on progress made in Europe. Since within the platform clinical, genetic, diagnostic, and therapeutic data of patients can be securely stored, another expected result is the creation of a “map” of neuromuscular patients across Europe.

Furthermore, the joint experience of experts who follow relatively large numbers of neuromuscular patients would also create a platform to develop recommendations on diagnosis, monitoring, and treatments. Finally, the shared knowledge and experience makes CPMS a potential ideal environment to select patients for future research activities: sharing clinical data, researchers will be in a facilitated position for the creation of ERN Registries and Databases and the selection of patients for ERN research projects (*e.g.* clinical trials). This remarkable and probably underestimated capacity that CPMS has to act as patients’ data repository should encourage further implementation, especially to maximize their interoperability with patients’ registries.

## Conclusions

The CPMS is an excellent tool and opportunity to enhance the service given to patients suffering with RDs. Thanks to the ability of the platform to securely store clinical, genetic, diagnostic, and therapeutic data of patients, the EURO-NMD CPMS has the potential to become an electronic health record database of neuromuscular patients across Europe.

Several approaches could be introduced or planned to promote the widespread use of the platform. Results presented in this paper clearly demonstrate that a tailored, ERN-oriented, training is a successful and promising strategy to significantly enhance the knowledge of CPMS and to implement its use in the daily practice. Similar courses should be designed through different ERNs in order to implement the use of CPMS and to benefit from strengths of e-health digital technologies.

Adaptations and improvements are expected to be applied the CPMS over the next years, in-line with the expressed necessities of the ERN community. To enhance the sustainability of the CPMS platform and to take fully advantage of its potential, it may be warranted to schedule case discussions via CPMS within each ERN network, so that panels can be periodically inserted and presented to the broader team in the network. As the volume of data entered in the CPMS increases, it will become strategic to take decisions on how to link such data with other data stored in separate repositories including registries of value to the ERNs’ sphere of expertise, electronic health records, biobanks, and more. Finally, it should be considered the person/month as effort the CPMS may benefit on, and possible modalities to grant that to HCPs. A support for CPMS usage will ensure usability and sustainability.

## Supplementary Information

**Additional file 1** The English version of CPMS consent form

**Additional file 2** Organization and structure of the training course

## Data Availability

The datasets generated during the current study are not publicly available due to CPMS restricted access to ERN members, but are available from the corresponding author on reasonable request.

## Abbreviations

CPMS: Clinical Patient Management System
ERN: European Reference Network
EU: European Union
GDPR: General Data Protection Regulation
HCP: Health Care Provider
MD: Medical Doctor
RD: Rare Disease

## References

[1] Website of the European Commission https://ec.europa.eu/health/non_communicable_diseases/rare_diseases_en. Accessed 15 July 2022

[2] Héon-Klin V (2017). European Reference networks for rare diseases: what is the conceptual framework?. Orphanet J Rare Dis.

[3] Website of the European Commission https://ec.europa.eu/health/ern_en. Accessed 15 July 2022

[4] Website of ERN EURO-NMD. https://ern-euro-nmd.eu/. Accessed 21 March 2023

[5] Deenen JC, Horlings CG, Verschuuren JJ, Verbeek AL, van Engelen BG (2015). The epidemiology of neuromuscular disorders: A comprehensive overview of the literature. J Neuromuscul Dis.

[6] Teoli D, Aeddula NR. Telemedicine. NCBI Books https://www.ncbi.nlm.nih.gov/books/NBK535343/ Accessed 15 July 2022

[7] Chirra M, Marsili L, Wattley L, Sokol LL, Keeling E, Maule S, Sobrero G, Artusi CA, Romagnolo A, Zibetti M, Lopiano L, Espay AJ, Obeidat AZ, Merola A (2019). Telemedicine in nurological disorders: opportunities and challenges. Telemed J E Health.

[8] Smith M, Alexander E, Marcinkute R, Dan D, Rawson M, Banka S, Gavin J, Mina H, Hennessy C, Riccardi F, Radio FC, Havlovicova M, Cassina M, Emandi AC, Fradin M, Gompertz L, Nordgren A, Traberg R, Rossi M, Trimouille A, Sowmyalakshmi R, Dallapiccola B, Renieri A, Faivre L, Kerr B, Verloes A, Clayton-Smith J, Douzgou S (2020). ERN ITHACA. Telemedicine strategy of the European Reference Network ITHACA for the diagnosis and management of patients with rare developmental disorders. Orphanet J Rare Dis.

[9] Smith CIE, Bergman P, Hagey DW (2022). Estimating the number of diseases—the concept of rare, ultra-rare, and hyper-rare. iScience..

[10] ERN CPMS https://cpms.ern-net.eu/login. Accessed 15 July 2022

[11] ERN CPMS https://ern-euro-nmd.eu/accessing-and-using-the-cpms/. Accessed 15 July 2022

[12] Anderson CL, Munawar S, Reilly L, Kamp TJ, January CT, Delisle BP, Eckhardt LL (2022). How functional genomics can keep pace with VUS identification. Front Cardiovasc Med.

[13] Monaghesh E, Hajizadeh A (2020). The role of telehealth during COVID-19 outbreak: a systematic review based on current evidence. BMC Public Health.

[14] Lepida platform https://www.lepida.net/. Accessed 15 July 2022

[15] Mönig I, Steenvoorden D, de Graaf JP, Ahmed SF, Taruscio D, Beun JG, Johannsen TH, Juul A, Hiort O, Pereira AM (2021). CPMS-improving patient care in Europe via virtual case discussions. Endocrine.

