# Supplementary material for: Digital health and Clinical Patient Management System (CPMS) platform utility for data sharing of neuromuscular patients: the Italian EURO-NMD experience
Source: Orphanet J Rare Dis. 2023 Jul 21;18:196. doi: 10.1186/s13023-023-02776-5 (PMC10360326; doi:10.1186/s13023-023-02776-5)
Supplement: Supplementary file 1 — Additional file 1 The English version of CPMS consent form [file 13023_2023_2776_MOESM1_ESM.pdf]

PATIENT CONSENT FORM FOR DATA SHARING
in
EUROPEAN REFERENCE NETWORKS FOR RARE DISEASES
for
PATIENT CARE and CREATION OF RARE DISEASE REGISTRIES

European Reference Networks

WHAT ARE THE EUROPEAN REFERENCE NETWORKS AND HOW CAN THEY HELP ME?

- European Reference Networks (ERNs) are networks of healthcare professionals working in rare diseases across Europe. They are established by Directive 2011/24/EU on the application of patients' rights in cross-border healthcare.
- ERNs exist to allow healthcare professionals to work together to support patients with rare conditions or other conditions which need highly specialized therapeutic procedures.
- With your consent, and in accordance with national and European data protection laws, your case may be referred to the ERN(s) named overleaf, so that the healthcare professionals in the ERN may help your doctor develop your diagnosis and care plan.
- In order for the ERN to advise on your care, the data collected about you in this hospital needs to be shared with healthcare professionals in other hospitals, some of which may be in other European countries. Your doctor can tell you more about which countries are in the ERN(s) relevant to your condition.
- Your care will remain the responsibility of the healthcare professionals who usually look after you.
- Data about you will not be shared without your consent, and even if you choose not to give your consent your doctors will continue to take care of you to the best of their ability.

PATIENT DATA SHARED FOR CARE WILL BE DE-IDENTIFIED

- If you and your doctors agree that it would be good to ask for support from one or more ERNs, this consent form will allow this hospital to share any of the data stored in your health care record which would help the healthcare professional in the ERN(s) to discuss your care.
- Your name and address will not be included.
- Such data may include medical images, laboratory reports, as well as biological sample data. It may also include letters and reports from other doctors who have cared for you in the past.
- If ERN(s) are consulted for your care, your data will be shared through a secure electronic information system called the ERN Clinical Patient Management System.

WHAT ABOUT RARE DISEASE DATABASES /REGISTRIES?

- To improve future knowledge on rare diseases, ERNs are very dependent on databases of information for research and knowledge development.
- Databases, also known as registries, contain only de-identified information. Your name, full date of birth or address are NOT included, only information about your condition.
- To help build the databases, you may give your consent for your data to be added to such databases. If you choose not give your consent this will not affect your care.

WHAT ABOUT RARE DISEASES RESEARCH?

- You may also let us know if you would like to be contacted about research projects for which your data could be used.
- If you agree to share your data for research you will be contacted to provide consent for a specific research project.
- Your data will not be used for research without your specific consent for a named research project.

WHAT ARE MY RIGHTS?

- You have the right to give or withhold your consent to sharing data in the ERN(s).
- If you consent today you may withdraw your consent later. Your doctor will explain how data about you can be removed from records if you wish. It may not be possible to remove information that has been used to care for you.
- You are entitled to receive further information about the purposes for which your data will be processed and who will have access to it. Your doctor can tell who can help you if you would like more information.
- You have a right to see which data is stored about you and to have corrections made to any errors you find. You may also have the right to block or erase your data.
- The hospital where your data is collected is responsible for your data. It should address your requests concerning your data in 30 days.
- It has the duty to ensure your data is processed safely and to notify you if a breach of data security occurs.
- If you have any concerns about the way in which your data is processed you may contact your treating doctor or your relevant data protection authority
- The need for keeping your data in the ERNs will be reviewed by your hospital every 15 years.

THIS CONSENT FORM MAY BE USED FOR SHARING DATA WITH THE FOLLOWING ERN(S)

(To be completed by the health care professional signing below)

……….

……….

……….

PATIENT DETAILS

First Name: ………………………………… **Surname:** …………………………………

Date of Birth: ☐☐ ☐☐ ☐☐☐☐ **ID number:** ☐☐☐☐☐☐☐☐☐

D D M M Y Y Y Y

Please tick the box that applies:

☐ **I am the patient** ☐ **I am the parent/guardian of the patient** ☐ **I have power of attorney**

I CONSENT to my de-identified data being shared in ERN(s) for my CARE

I understand that my data will be shared with healthcare professionals in the ERN (s) so that they may work together to support my care.

Signature **Date**

………………………………… …………

I DO NOT CONSENT to my data being shared in ERN(s) for my CARE

I understand that this means the ERN(s) cannot be consulted to support my care.

Signature **Date**

………………………………… …………

I CONSENT *to my de-identified data being included in one or more ERN database or registry.*

Signature **Date**

………………………………… …………

I DO NOT CONSENT *to my data being included in an ERN database or registry.*

Signature **Date**

………………………… …………

I WOULD LIKE TO BE CONTACTED *about research. I will decide if I consent to my data being used for a specific project if I am contacted.*

Signature **Date**

…………………………………. …………

I DO NOT WANT TO BE CONTACTED *about my data being used in research.*

Signature **Date**

………………………… …………

TREATING PHYSICIAN or PERSON AUTHORISED TO WITNESS CONSENT

Name ……………………………………………. **Position** ……………………………………… **Date** ……………

WHERE CAN I FIND MORE INFORMATION?

You can also find more information about ERNs at

https://ec.europa.eu/health/ern_en
