# Supplementary material for: Digital health and Clinical Patient Management System (CPMS) platform utility for data sharing of neuromuscular patients: the Italian EURO-NMD experience
Source: Orphanet J Rare Dis. 2023 Jul 21;18:196. doi: 10.1186/s13023-023-02776-5 (PMC10360326; doi:10.1186/s13023-023-02776-5)
Supplement: Supplementary file 2 — Additional file 2 Organization and structure of the training course [file 13023_2023_2776_MOESM2_ESM.docx]

Organization and structure of the training course

Implementation of the training

The training course took place remotely, and was divided in two parts detailed below. The first part of the training lasted four months (from September 2020 to December 2020) with six lessons per month of approximately two hours each. The second part of the training lasted five months (from February 2021 to June 2021), with four lessons per month of approximately two hours. The entire schedule of the training was adjusted on clinical activities of the attendees, to ease course attendance without interfering with other duties.

First phase of the training

The training started with the explanation of the course goals and aims and with a delineation of a course syllabus. The trainers showed the CPMS general functioning and its main applications, and provided training materials on the platform's use.

In the initial phases of the training, a training version of the CPMS platform had been used to display the whole procedure, from patient enrolment until panel closure. The training version of the platform is so that dummy data and patients can be added without the risk of interfering with real data. When participants were ready for discussing their own patients, trainers encouraged participants in using their own real platform to enroll real patients.

At that time, the course focused on the first steps needed for case discussion. In particular, all the procedure of patients’ enrollment and of the panel opening were elucidated, and much time was devoted in filling the consultation form (the core of case description). In the consultation form is possible to provide all the information about a clinical case, from blood tests to clinical examinations and is possible to add clinical reports as well. Is even possible to detail a family history attaching a pedigree and is also possible to attach imaging studies’ reports and imaging data.

After these first steps, the clinician that is opening the panel is supposed to invite members from other Centers for panel discussion. Attention was thus paid on all the steps deemed necessary to invite members and to schedule video meetings for panel discussion. Particularly, video meetings have been held in the upcoming lectures in order to ease both the panel discussion from a medical point of view and the use of the video meeting and the several tasks related. In parallel, trainees carried on opening new panels, while the training course moved forward following the panel timelines. At this point, the course focused on the final steps of the panel timeline, until panel closure.

Second phase of the training

The second part of training course was fully interactive and focused on panel progression monitoring. Trainees were supported in progressing opened panels and were encouraged in opening new panels. Case discussions were held during these lessons to ease panels progression. The training course ended in June 2021.
